# Continuous-Spectrum Infrared Illuminator for Camera-PPG in Darkness

**DOI:** 10.3390/s20113044

**Published:** 2020-05-27

**Authors:** Wenjin Wang, Luc Vosters, Albertus C. den Brinker

**Affiliations:** 1 Philips Research, 5656 AE Eindhoven, The Netherlands; bert.den.brinker@philips.com; 2 Philips Intellectual Property & Standards, 5656 AE Eindhoven, The Netherlands; luc.vosters@philips.com

**Keywords:** vital signs monitoring, photoplethysmography, biomedical sensing, camera, near infrared, illumination

## Abstract

Many camera-based remote photoplethysmography (PPG) applications require sensing in near infrared (NIR). The performance of PPG systems benefits from multi-wavelength processing. The illumination source in such system is explored in this paper. We demonstrate that multiple narrow-band LEDs have inferior color homogeneity compared to broadband light sources. Therefore, we consider the broadband option based on phosphor material excited by LEDs. A first prototype was realized and its details are discussed. It was tested within a remote-PPG monitoring scenario in darkness and the full system demonstrates robust pulse-rate measurement. Given its accuracy in pulse rate extraction, the proposed illumination principle is considered a valuable asset for large-scale NIR-PPG applications as it enables multi-wavelength processing, lightweight set-ups with relatively low-power infrared light sources.

## 1. Introduction

Camera-based remote photoplethysmography (remote-PPG) enables contactless measurement of the cardiac pulse by detecting the pulse-induced subtle color changes from the human skin surface [1]. The implementation of remote-PPG in near-infrared (NIR) promises attractive applications in darkness, as it involves invisible light that is less obtrusive to the human eye. NIR-PPG is particularly relevant for patient monitoring in high-acute settings in hospitals that require long-term continuous monitoring (24/7), such as in Intensive Care Units (ICU) [2] and Neonatal Intensive Care Units (NICU) [3]. It is also desired for low-acute settings or free-living applications, such as baby/elderly care at home [4] and driver monitoring [5] in automotive. Especially for automotive, it requires the complete remote-PPG solution, including camera and light source, to be energy efficient, lightweight (for integration), and cost-effective (for large-scale deployment).

Multispectral cameras, including the RGB camera (adapted to NIR) used in this study, play an essential role in robust pulse-rate monitoring [6]. There are two main benefits of multi-wavelength sensing over single-wavelength sensing (by monochrome cameras). First, since blood pulsation has a characteristic amplitude profile on different wavelengths in contrast to signal disturbances caused by motion or illumination changes, multispectral cameras allow us to extract the cardiac signal components while suppressing the signal disturbances. Various PPG-extraction algorithms [6,7,8,9,10,11,12,13] proposed to address motion challenges profit from the fundamental use of multi-wavelength measurements. Second, it enables the measurement of other physiological variables in addition to the pulse rate and thus it can potentially extend the scope of vital signs monitoring, such as blood oxygen saturation which requires simultaneous measurement of at least two infrared wavelengths.

**Figure 1:**
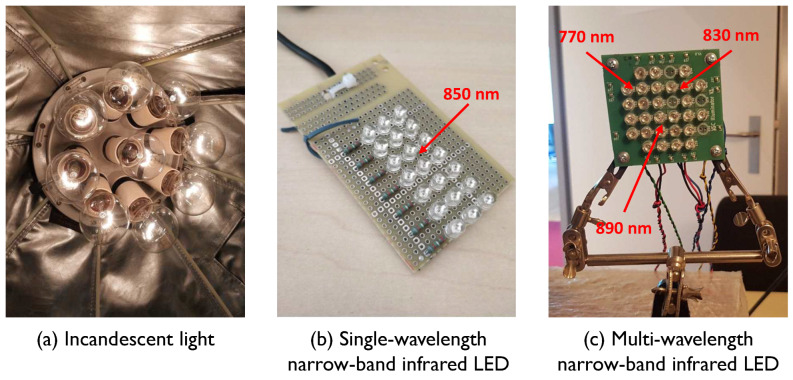
Different light sources that could be considered as the illumination unit of an infrared remote-PPG setup.

In view of the advantages of infrared measurement and multispectral sensing, we advocate for a multi-wavelength infrared setup for camera-based vital signs monitoring. A multi-wavelength NIR-sensing setup requires both the camera sensor and light source to be multispectral in infrared. While solutions for multi-wavelength NIR cameras have been studied (e.g., RGB2NIR system [14,15] and multi-camera system [11,16,17]), a proper light source for NIR-PPG has received much less attention. Thus far, three types of light source for NIR-PPG illumination have been reported (see Figure 1):

(i) *Incandescent light*: It provides a continuous spectrum in infrared, which has been used for proof-of-concept validation of the multi-wavelength NIR setup [11,16,17]. It is obviously not suitable for real use cases (e.g., automotive and care units in hospital) due to the produced visible light and heat. Although this visible part can be blocked, the system remains bulky and is not energy-efficient. In addition, incandescent lights are not available for purchase anymore in many countries. Hence, incandescent light is not in our consideration for applications and products.
(ii) *Single-wavelength narrow-band infrared LEDs*: A panel of infrared LEDs can provide a single and narrow-band illumination in infrared and this is commonly used for night-vision surveillance in combination with monochrome cameras. It has also been applied in the context of single-wavelength NIR-PPG measurement [18,19]. Its performance has been benchmarked with the multi-wavelength approaches in earlier studies [11] and has clearly shown a worse performance. The fundamental limitation is that it has no additional wavelength-channel information for suppressing non-cardiac signal disturbances caused by motion or illumination changes, and it cannot be extended to measure other vital signs (e.g., blood oxygen saturation) that require multispectral sensing. Thus, it is not a favorable option for wide-spread application.
(iii) *Multi-wavelength narrow-band infrared LEDs*: A panel of infrared LEDs using narrow-band infrared LEDs at various wavelengths has the problem of spectral color inhomogeneity in the emitted light. This is because different LEDs have different spatial locations and thus have different illumination directions that emphasize different parts in an image (i.e., inhomogeneity). The intensity ratios of these narrow-band wavelengths depend on the pixel location in an image. Thus, subject movement causes “spectral color changes” on the skin surface. As a result of spectral color inhomogeneity, the exact spectral components measured on the skin surface are disturbed by body motion. Model-based disturbance reduction methods [6,9,10] cannot handle that adequately, nor can data-driven methods [7,8,20], since the color variation directions (e.g., blood volume pulse signature [10]) vary continuously over the motion sequence. An experimental verification of its inhomogeneity is given in Section 2.1.

In this work, we focus on the illumination unit and advocate a multi-wavelength NIR illuminator using phosphor LED. It is the first time for the phosphor LED to be used as the active illuminator for NIR-PPG. Phosphor LED is characterized by its continuous spectrum in infrared, low power consumption, and is an option for devices requiring a lightweight design. These factors are necessary conditions for the introduction of large-scale NIR-PPG applications. The developed illumination unit was tested in a multi-wavelength NIR setup (with an RGB2NIR camera [14] and an end-to-end software processing unit [11]). The complete solution is a first of its kind and has been validated in darkness with subject body motions and shows a robust performance of pulse-rate measurement.

The remainder of this paper is structured as follows. In Section 2, we describe different components in a multi-wavelength NIR setup with focus on the light source unit (Phosphor LED). In Section 3 and Section 4, we use a benchmark dataset deploying a practical experimental protocol to verify the proposed solution using state-of-the-art combinations of camera and processing algorithms together with the developed light source. Finally, in Section 5, we draw the conclusions.

## 2. Materials and Methods

This section presents the materials and methods that are essential for building the multi-wavelength NIR setup. Firstly, we demonstrate the color inhomogeneity effect in case of multiple narrow-band LEDs illumination. The next three subsections are devoted to the three fundamental building blocks of the considered system: the illumination, camera, and processing unit.

### 2.1. Challenge of Multi-Wavelength Narrow-Band LEDs

Consider a multiple narrow-band LED light source, as shown in Figure 1c; it consists of a panel of 4 pieces of 770 nm, 12 pieces of 830 nm and 16 pieces 890 nm narrow-band LEDs that are interleaved across the PCB. The number of LEDs for each particular wavelength is different because the camera quantum efficiency varies per wavelength. In particular, silicon-based cameras are more sensitive to lower wavelengths such as 770 nm than higher wavelengths such as 890 nm. Therefore, more LEDs of 890 nm than 770 nm are needed to attain a balanced DC across the camera channels when viewing the skin tissue. To get a smooth output and facilitate the mixing of wavelengths, we added a Markolon diffuser [21] in front of the LEDs.

**Figure 2:**
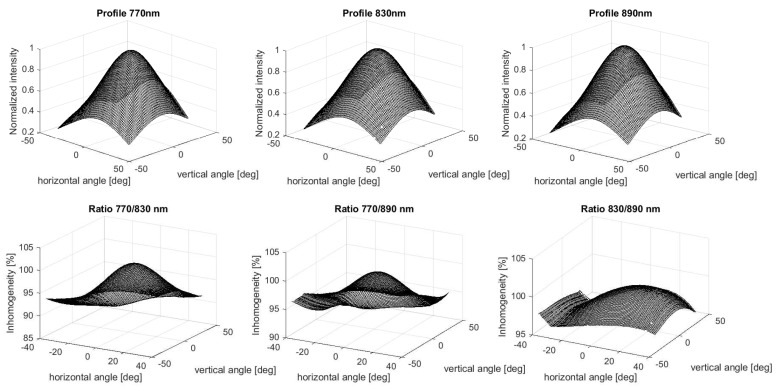
Color homogeneity measurements of the multi-wavelength narrow-band LED light source of Figure 1c (with diffuser). The measurements were done in a dedicated light box with an optical power meter.

We tested the color homogeneity of this light source, and also executed a pilot experiment. Figure 2 shows the normalized intensity and the ratio of intensities among the three different wavelengths as a function of the horizontal and vertical angles. The measurements were done in a light box with a rotating actuator on which the light source was mounted. The actuator was panned from −40 to 40 degrees and tilted from −40 to 28 degrees with a step size of 1 degree. For each step, we measured the intensity of the 770, 830 and 890 nm LEDs independently, normalized it, and plotted it in the first row in a 3D view. The bottom row shows the intensity ratios between all wavelengths. A constant ratio means that the light source exhibits a perfect color-homogeneity over the illumination space.

Figure 2 shows that the color homogeneity of the light source varies from 90% to 100% over the measured angular range. The fundamental problem caused by color inhomogeneity is that the intensity distribution for each narrow-band wavelength is different. Consequently, the composition of the spectrum is different locally and depends on the relative position and angle of the skin with respect to the light source. Moreover, the spectrum measured on the skin varies due to body motion, depending on the speed, direction, and level of the motion. Since the color changes induced by blood pulsation is a vital sign, the color changes resulting from motion will significantly disturb the PPG extraction.

We note that local changes in the spectrum are more problematic than shadows. Shadows do not cause local changes in the color spectrum, merely an intensity change, which can be addressed by the existing PPG-extraction algorithms through multi-wavelength combination/projection [6] (i.e., be orthogonal to the intensity variation direction). In contrast, color inhomogeneity gives rise to the uncertainties and variations of pixel spectrum that fundamentally change the characteristics of blood absorption variation in different wavelengths, which jeopardizes the PPG extraction.

**Figure 3:**
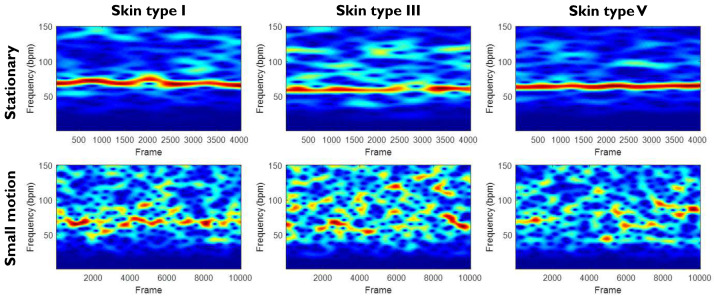
The pilot measurement of PPG spectrograms measured from three subjects (with different skin types) in two different scenarios (stationary and small motion). The light source was the multi-wavelength narrow-band LED light source configuration shown in Figure 1c including a diffuser.

Notwithstanding the large color inhomogeneity, we did a pilot experiment with the designed system to investigate its behavior (or feasibility). Figure 3 shows the pilot measurement of three subjects with the multi-wavelength narrow-band LED light source we constructed in Figure 1c. The three test subjects have different skin types (Type I, III, and V) according to the Fitzpatrick scale. Each subject was recorded in two scenarios: stationary (no voluntary motion) and small motion, where “small motion” is defined based on the statistical range of four motion categories (e.g., stationary, small, medium, and large) quantified in our benchmark dataset that will be introduced in Section 3. Empirically, we found that the quality of measurements (e.g., pulse spectrograms) is rather poor when subjects have small body movement. This is in line with the verification of color inhomogeneity in Figure 2. The obvious way of improving the color homogeneity would be to increase the distance between the skin and light source. However, this solution is not feasible in many applications (e.g., automotive and neonatal incubator). We also looked into various alternatives to improve the color homogeneity, such as wide-angle diffusers, mixing chambers, vertical cavity surface-emitting lasers, microlens arrays, etc. In view of the result, we need a significant improvement on the setup to reduce the color inhomogeneity, yet perfect color homogeneity (i.e., 0% inhomogeneity) is impossible to achieve. The modifications on the hardware are also difficult to implement due to practical restrictions/considerations, such as the dimension of a single LED element, form factor of the required optics, overheating when densely integrating multiple LEDs, cost of implementation, quality of processing technology, etc. Based on the homogeneity measurement and pilot experiment, we argue that multi-wavelength narrow-band LED light sources are not well suited for remote-PPG and decided not to pursue this option for further development. We also did not include it in our later experiments as the expected performance is below any reasonable standard.

### 2.2. Illumination Unit

**Figure 4:**
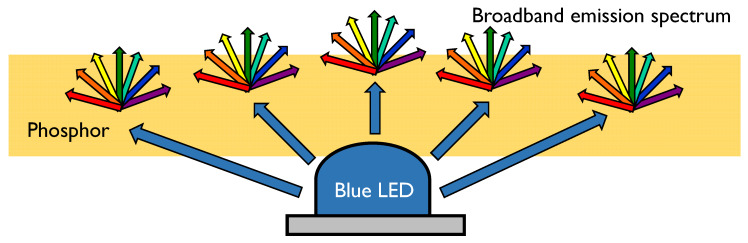
Working principle of a broadband LED emitter [23]: a blue LED is used to illuminate a piece of phosphor which then emits a wide spectrum. Some of the original blue light also goes through.

We consider phosphor to be the ideal material for making a continuous-band LED. When phosphor is illuminated by a deep blue (ultra-violet) light source, it emits visible light. The spectrum of this emitted light can be controlled by tuning the phosphor’s composition, structure, and size. Due to this flexibility and control, phosphors are used extensively to manufacture displays, fluorescent lighting, and white LEDs [22]. Figure 4 illustrates how a phosphor LED works. A phosphor layer is stacked on top of a blue LED. The LED excites the phosphor, which then emits light with a broad spectrum.

The main application of phosphor LED is spectroscopy. In spectroscopy, an object is illuminated with a broad spectrum light source. The reflected spectrum, which is different for various materials since each material absorbs different wavelengths, is then measured with a camera, spectrometer, or wavelength sensitive detectors and compared to known spectra in a database. From this comparison, the type of object and/or composition of the object can be identified. Due to recent technological advances, the cost and size of the optics for spectroscopy have significantly reduced. It is expected that in the near future it will be possible to integrate a spectrometer (including phosphor LED) in a mobile phone, paving the way for portable physiological measurement (pulse rate, blood oxygen saturation, hydration, etc.).

The emission spectrum of phosphor LED has traditionally been limited to cover a range of 450–700 nm. More recently, phosphor LEDs with a broader spectrum have been introduced to the market. For example, the Lumileds’ LUXEON IR ONYX expands the usable wavelength range into near infrared, adding 700–1050 nm and beyond [23]. This extended wavelength range makes it possible to use phosphor LEDs for monitoring a subject’s pulse rate with a vital signs camera (infrared camera) in darkness. The main benefit of phosphor LED light sources compared to light sources consisting of multiple narrow-band LEDs with various wavelengths is its excellent color homogeneity properties, meaning that at every point on an illuminated surface the light spectrum is identical. Consequently, these light sources will achieve better motion robustness in pulse-rate monitoring, as compared to the illuminator made of multi-wavelength narrow-band infrared LEDs (Figure 1c).

**Figure 5:**
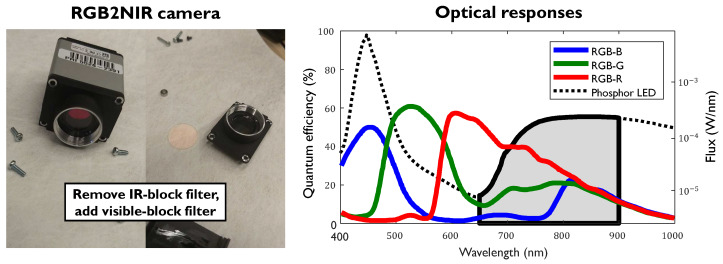
The multi-wavelength NIR camera unit RGB2NIR: (**Left**) the re-configuration of the RGB camera; and (**Right**) camera sensitivity profiles (red, green, and blue), illumination spectrum (dashed), and IR-passed spectral components (solid black line with gray area). The left y-axis denotes the quantum efficiency of camera, and the right y-axis denotes the Flux of Phosphor LED.

**Figure 6:**
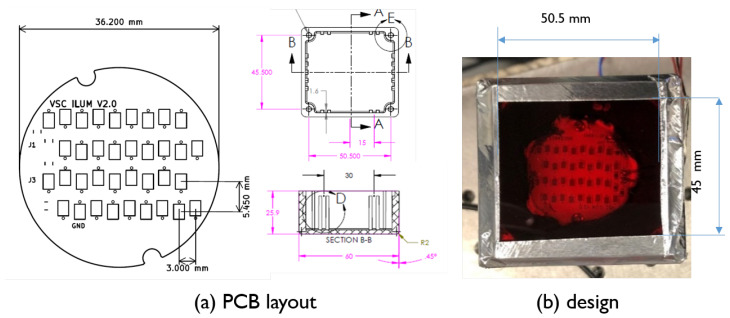
Details of the constructed illumination unit: (**a**) PCB drawing; and (**b**) final design of our built illuminator (with a visible light block filter).

We built a prototype light source with the Lumileds’ LUXEON IR ONYX phosphor LEDs [24] to test the feasibility for the use in a vital signs camera. The spectrum of this LED is shown in Figure 5. This figure shows that, although there is significant light power in the range of 650–1050 nm, a strong peak of blue light at 450 nm is also present. The optical output power in this peak is roughly 100 times stronger than the maximum peak in the near-infrared range. Hence, we use a long-pass blocking filter with a cutoff at 650 nm (see Figure 5) to suppress this peak. The LEDs are mounted on a PCB, as shown in Figure 6a, in strings of 4 by 9. The current through each string is 367 mA and the voltage across each string is 30 V. The total power consumption of the light source is 44 W. The PCB is encapsulated in an aluminum box of dimensions 60 × 50 × 25.9 mm of which the inside is coated black (see Figure 6b) to prevent blue light from escaping and to adhere to all eye-safety requirements (according to IEC 62471 Photobiological safety of lamps and lamp systems).

Due to technical limitations, we cannot make the same color homogeneity analysis as in Figure 2 for our proposed light source built from Phosphor LEDs. The reason is that Phosphor LEDs emit a continuous spectrum where individual wavelengths cannot be switched on or off. Therefore, we cannot measure the intensity of each wavelength individually as we did in the measurement setup for the multi-wavelength narrow-band LED light source. However, we note that each phosphor LED is a point light source that emits exactly the same spectrum in every direction. Hence, we argue that the color homogeneity should be very constant across the illuminated space.

### 2.3. Camera Unit

As the goal of this study is to build a practical setup for NIR-PPG monitoring, we choose the RGB2NIR system [14] for the camera unit. Therefore, we adapt an RGB camera to the NIR camera by replacing its IR-block filter with a visible-block filter. The RGB camera used for adaption is: IDS UI-3200SE-C-HQ (Sony IMX304LQR-C, CMOS sensor, global shutter). The visible-block filter passes 650–900 nm wavelengths.The camera lens is: Tamron 1.1 50 mm F/1.8 (Model M111FM50). The spectra of the camera and illumination combined with the visible block filter are shown in Figure 5.

### 2.4. Processing Unit

**Figure 7:**
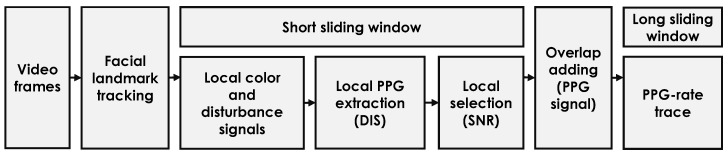
The architecture of the processing unit used for end-to-end pulse-rate extraction from a video [11].

To measure the pulse-rate from a video, we used an existing camera-PPG extraction architecture (see Figure 7) introduced in [11]. The major algorithmic processing steps include: a facial landmark tracker [25], a short sliding window for extracting the color and motion signals from local skin patches defined by the facial landmarks, a core PPG-extraction algorithm (DIS [11]) with a band-pass filter to extract local PPG signals, a Signal-to-Noise-Ratio (SNR)-based metric to select the local PPG candidates for global combination, and an overlap-add procedure to generate a long PPG signal based on the short PPG intervals measured per sliding window. In the end, we use a long sliding window to generate the pulse-rate trace from the measured PPG signal, where the pulse rate is calculated as the frequency index of the maximum spectral peak in the frequency domain. We stress that, since this study is focused on the exploration and validation of the light source (not the algorithmic solution), all the parameters (e.g., facial landmarks, window length, and DIS coefficients) used in video processing are kept the same as those in [11] (i.e., default setting).

## 3. Benchmark Protocol

This section presents the benchmark protocols. We first introduce the benchmark dataset, and then the evaluation metrics.

### 3.1. Benchmark Dataset

We built a video dataset to verify the proposed illumination unit. It includes a total of 17 healthy adult subjects (aged between 25 and 55 years), with different skin types categorized from Type I to VI based on the Fitzpatrick scale. This study was approved by Philips Intellectual Property & Standards, and written informed consent was obtained from each participant. As for the recording setup, the camera and the proposed light source were placed on a tripod, which was around 2 m in front of the subject. The subject sat on a chair with his/her face recorded. With the used camera focal length, the skin area accounts for 30–40% of pixels per video frame. The videos were recorded in uncompressed format (with 1280×960 pixels, 8 bit depth) at a constant frame rate (15 frames per second). All the auto-adjustment functions of the camera, e.g., auto-focus, auto-gain, auto-white-balance, and auto-exposure, were turned off during the recording.

The experimental protocol was designed as follows. Each video recording has seven phases: four stationary phases with three motion phases in between. Each phase was approximately 1 min long. In the odd-numbered phases, the subject remained stationary (no voluntary motions). In the even-numbered phases, the subject was instructed to perform free head movements and facial expressions, resulting in a mixture of rotation, translation, scaling, smiling, and talking. In those motion phases, the subject was also instructed to mimic different levels of motion. We categorize the motions in Phases 1, 2, and 3 as “small motion”, “medium motion”, and ”large motion” respectively.

**Figure 8:**
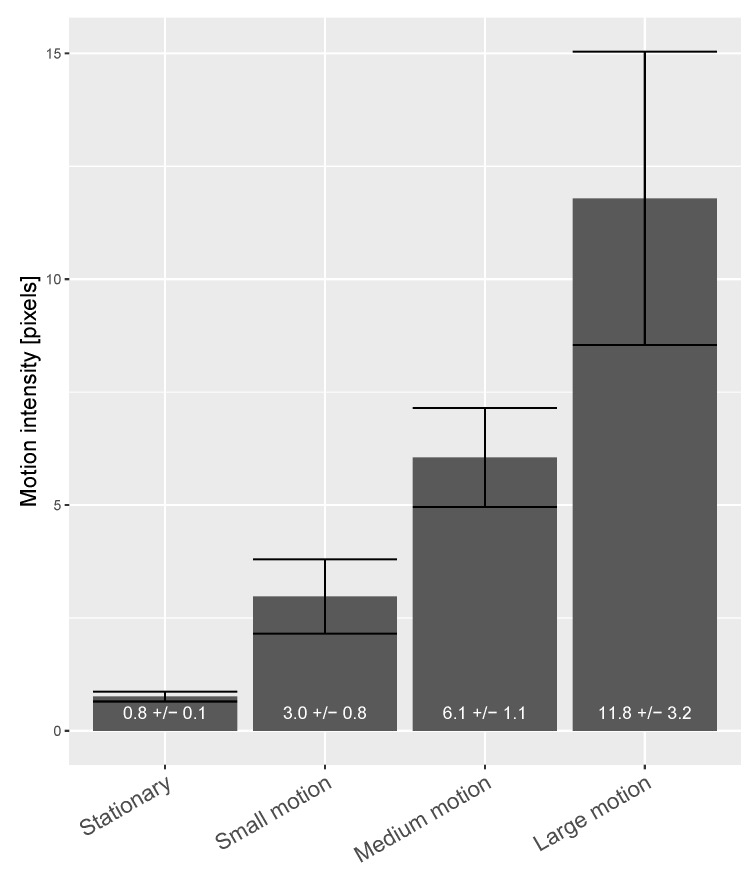
Statistical overview (mean and 95% confidence interval) of HR-band motion intensities of 17 test subjects, categorized in terms of stationary, small motion, medium motion, and large motion.

To quantify the four motion scenarios of a subject in the recording (i.e., stationary, small motion, medium motion, and large motion), we used a metric, called “HR-band motion intensity”, to measure the significance of motion that particularly disturbs the PPG extraction. The metric takes the averaged facial landmark trace (i.e., motion trace) as the input, and post-processes it by removing the distortions outside the heart-rate band as this part does not interfere pulse measurement. The post-processing is done by the same band-pass filter used for remote-PPG (i.e., the fourth-order Butterworth band-pass filter with cutoff frequencies [0.6, 3] Hz in a zero-phase forward and reverse filtering mode). Then, it applies the same sliding window as used for calculating the pulse-rate, to calculate the motion standard deviation, which represents motion intensity/energy inside the heart-rate band (with the unit “pixel”). We quantified the motion intensities for 17 test subjects based on the recording protocol. Figure 8 shows the average HR-band motion intensity metric for each motion category (“small motion”, “medium motion”, and “large motion”) of the benchmark protocol.

A finger-based transmissive pulse oximeter (Model: Philips IntelliVue X2) was used as the reference to collect the ground-truth. The device was electrically decoupled from the video recording system. The raw PPG signal from the finger oximeter was collected and synchronized with the video acquisition using time stamps. The reference signal was post-processed by a band-pass filter (i.e., the fourth-order Butterworth band-pass filter with cutoff frequencies [0.6, 3] Hz in a zero-phase forward and reverse filtering mode) to reduce the outliers induced by occasional finger motions.

### 3.2. Evaluation Metrics

We used two metrics (availability and root mean square error) to investigate the performance of pulse-rate extraction in the framework of Receiver Operating Characteristic (ROC) curve.

- **Availability**. We used availability because it has been adopted by the Consumer Technology Association (CTA) as a standard metric in the industry for heart-rate monitoring [26]. It is also a key concept in communication with customers/manufactures (e.g., fitness and automotive). The availability is given as:
  (1) $$A\left(e\right)=\frac{\sum_{i=1}^{L}b_{i}\left(e\right)}{L}\;\mathrm{with}\;b_{i}\left(e\right)=\left\{\begin{array}{cc} 1, & \mathrm{if}\;|\Delta_{i}|\leq e\\ 0, & \mathrm{otherwise} \end{array}\right.,$$
  where $|\Delta_{i}|$ denotes the absolute difference between the reference PPG-rate and camera PPG-rate at the time index *i*; *e* is the tolerance error (i.e., e=2 means allowing 2 bpm difference); *L* is the total number of pulse-rate measurements (i.e., length of the PPG-rate trace); and A(e) counts the fraction of pulse-rate measurements that are deviating less than *e* bpm from the ground-truth. Larger A(e) suggests better measurement coverage.
- **Root Mean Square Error (RMSE)**. We used RMSE to measure the difference between the contact-based reference PPG-rate trace and camera PPG-rate trace within the specified error range *e*. RMSE, denoted as σ, is given by:
  (2) $$\sigma \left(e\right)=\sqrt{\frac{\sum_{i=1}^{L}b_{i}\left(e\right)\cdot {\left|\Delta_{i}\right|}^{2}}{\sum_{i=1}^{L}b_{i}\left(e\right)}}.$$
  RMSE was used as the accuracy metric in this study. Smaller σ(e) suggests more accurate PPG measurement.
- **ROC curve of Availability and RMSE**. As for the statistical analysis, we plot RMSE (σ), as a function of availability (*A*), for e∈{1,2,...,10,15,20,30,Inf} bpm in an ROC curve, where the *x*-axis denotes availability and the *y*-axis denotes RMSE. The benefits of such an ROC curve is to serve the readers/users from different research areas or with different application backgrounds. For clinicians and medical doctors in an ICU environment, a high accuracy is essential while the requirements on availability are more relaxed. Their focus is on lowering the false positive rate. This can be achieved by allowing fewer false positives by accepting a lower availability. On the other hand, manufacturers of assisted-living applications (fitness or automotive) have more stringent requirements on availability compared to RMSE. They aim at a rough estimate of heart rate and can be more tolerant to false positives. An ROC curve is an excellent tool for visualizing the trade-off between availability and RMSE. In addition, it gives a complete view on the full MAE and Availability ranges that can be achieved. This leads to a more fair and complete benchmark. Moreover, the ROC curve is an essential tool for designing a non-reference based control mechanism, i.e., quality metric, that can be used to tune a system or algorithm to a desired operating point on the curve. We consider quality metrics to be beyond the scope of this study.

## 4. Results and Discussion

This section presents the benchmark results and analysis. In Section 2, we demonstrate that light sources built from multi-wavelength narrow-band LEDs have poor color homogeneity. Figure 3 shows that it cannot even attain a reasonable performance in a scenario with small motion. Thus, we did not include this type of light source in the benchmark intended for motion robustness. We compared the proposed Phosphor LED setup with the incandescent light source that has been prototyped/validated for NIR-PPG measurement [11]. We stress that, since we cannot incorporate two different light sources in the same video recording for a synchronous comparison, we have to compare to the results obtained in a different study with the incandescent light source and such comparison will be on a qualitative level. In particular, we refer to the benchmark of Wang et al. [11] that was performed by the same authors in the same environment (e.g., same lab) on similar subjects. Hereby, we add a description to clarify the settings of the incandescent light source in [11]. This light source consists of two incandescent light fixtures with nine lamps each. Each fixture is powered at 220 V × 1.2 A = 264 W, which supplies sufficient energy for the NIR sensing. Two fixtures are placed on the two sides of the subject with 45${}^{{}^{\circ}}$ angle, providing a homogeneous illumination condition. For the current experiment, we replicated the whole procedure to enable a direct comparison with the results in [11].

**Figure 9:**
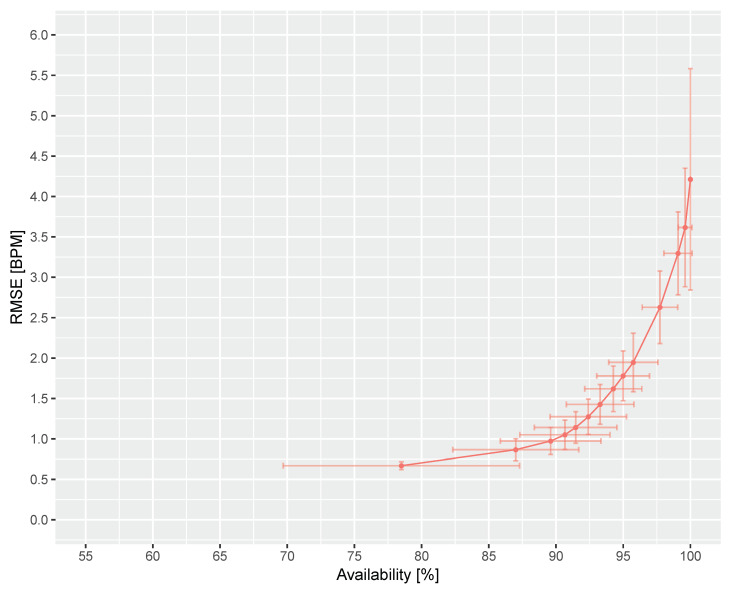
ROC curve of DIS in the benchmark dataset. The scatter denotes the RMSE at a specific availability. The horizontal and vertical bars denote the 95% confidence ranges of availability and RMSE, respectively.

Figure 9 shows the ROC curve for the complete benchmark dataset including the 95% confidence interval on availability and RMSE. As can be seen, the RMSE is smaller than 1 bpm when the availability is smaller than 90%. It also suggests that, when the availability is small (i.e., more restrictive to large errors), its uncertainty is large but the RMSE will be small. When the availability is large (i.e., more tolerant to large errors), its uncertainty is small but the RMSE and the RMSE uncertainty will be large. As mentioned above, the ROC curve in Figure 9 can be use to design a non-reference based quality metric to reject large errors and, given application specific requirements on RMSE and availability, it allows tuning to a desired operating point on the curve.

**Figure 10:**
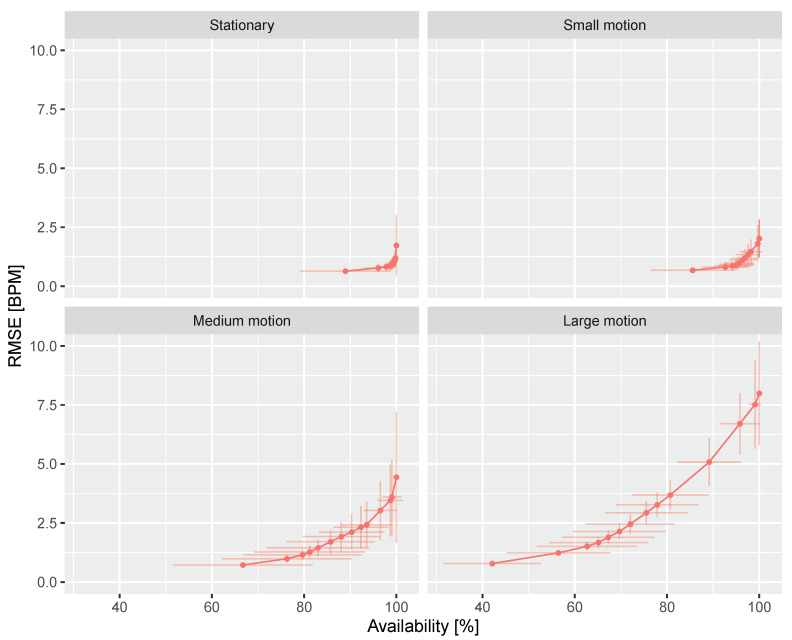
ROC curve of DIS in four categorized scenarios of the benchmark dataset. The plotting conventions are equal to those in Figure 9.

Figure 10 shows the ROC curve of DIS in four categorized scenarios with different amounts of motion. It is in line with our expectation that significant body motions deteriorate the measurement. For the stationary scenario (i.e., typical use case of sleep monitoring), its RMSE is smaller than 2 bpm at 90% availability. For the large motion scenario (i.e., automotive), its RMSE can be smaller than 2 bpm when the availability is chosen around 65%.

Table 1 summarizes the RMSE (with confidence interval) of DIS at 100% availability averaged over all participants and phases of the recording (e.g., motion and stationary). The benchmark recordings have been categorized into four scenarios (i.e., large motion, median motion, small motion, and stationary) based on the significance of motion. In the complete benchmark dataset, DIS has an RMSE of 4.00 ± 1.07 bpm, which is comparable to what we obtained with an incandescent illuminator for NIR-PPG [11]. In more details, Wang et al. [11] reported an RMSE less than 2 bpm for the stationary scenario and an RMSE in the range of 5–10 bpm for motion scenarios (depending on the level of motion). Table 1 clearly shows that, with the proposed light source, we attain a performance in the same range.

**Figure 11:**
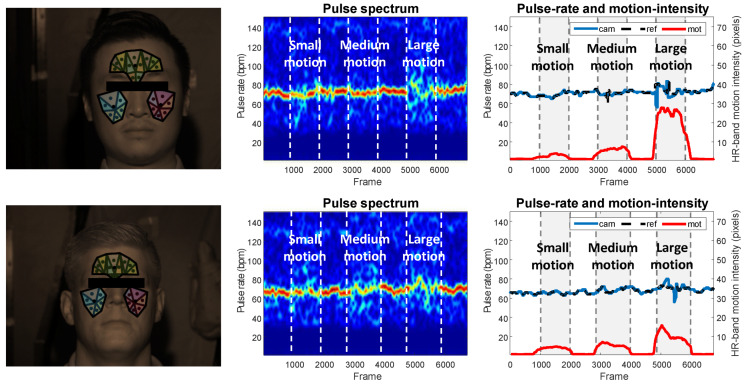
Exemplified measurements obtained by the processing flowchart with the DIS algorithm. From left to right: video frames showing the measured skin areas, pulse spectrogram, and pulse-rate and motion-intensity traces. The x-axis denotes the frame number (time axis); the left y-axis denotes the pulse rate (frequency axis); and the right y-axis denotes the HR-band motion intensity (pixel axis). The reference PPG-rate is denoted by the dashed black line. The motion periods are indicated by gray padding. The HR-band motion intensity is denoted by the red line (to indicate the significance of motion).

Figure 11 exemplifies the PPG spectrograms and PPG-rate traces of two test subjects in the benchmark. According the protocol, there are three epochs of continuous motion in each video recording, annotated by the gray padding in the figure. As can be seen, the camera measured PPG-rates are well-aligned with the contact reference, even in the motion periods with significant head movements. It implies that the designed multi-wavelength illumination provides sufficient characteristics that are needed for the multi-wavelength PPG extraction algorithm to distinguish pulse from motion.

From preliminary experiments on the prototyped light source, we found that, when the distance between the light source and test subject is reduced to 0.5 m, a realistic scenario for many applications in particular driver monitoring and home care, only 12 LEDs are required at a total power consumption of 8 W. As for the future work, we will continue working on an integrated NIR-PPG solution based on the investigated Phosphor LED, which will be low-cost, low-power, lightweight, and with a compacter form factor. We will validate it and demonstrate its performance in practical scenarios such as automotive and clinical units (e.g., ICU and NICU).

## 5. Conclusions

In this paper, we introduce a new continuous-spectrum infrared illuminator for remote pulse-rate measurement in darkness. It solves the limitations of previous infrared light sources (e.g., incandescent light, narrow-band infrared LED, etc.) for this application. Our proposed light source is based on the principle of Phosphor material that emits a continuous broadband spectrum in the infrared range. A first prototype of the light source was designed and implemented. It was demonstrated within a remote-PPG monitoring scenario in darkness, on the subjects with body motions. The promising results suggest that the proposed multi-wavelength infrared illuminator is a favorable option for a broad range of applications/products in camera-based vital signs monitoring.

## Figures and Tables

**Table 1:** The σ obtained by DIS at 100% availability using averaging over all participants and recording phases. The 95% is the confidence interval, i.e., if repeating the same measurement, RMSE will be between σ±C with 95% probability.

Evaluation Metric	Large Motion	Median Motion	Small Motion	Stationary	Complete
σ (bpm)	7.99	4.44	2.02	1.73	4.00
*C* (bpm)	2.20	2.77	0.80	1.26	1.07

## Abbreviations

The following abbreviations are used in this manuscript:

PPG	Photoplethysmography
NIR	Near Infrared
RMSE	Root Mean Square Error
ROC	Receiver Operating Characteristic
ICU	Intensive Care Units
NICU	Neonatal Intensive Care Units
